# Response to Immune Checkpoint Inhibitor Treatment in Advanced Cervical Cancer and Biomarker Study

**DOI:** 10.3389/fmed.2021.669587

**Published:** 2021-08-12

**Authors:** Kevin R. Shieh, Anna Huang, Yiqing Xu

**Affiliations:** ^1^Department of Medicine, Maimonides Medical Center, Brooklyn, NY, United States; ^2^Comparative Effectiveness Outcomes Research, Department of Sociomedical Sciences, Columbia University Mailman School of Public Health, New York, NY, United States; ^3^Division of Hematology and Oncology, Department of Medicine, Maimonides Medical Center, Brooklyn, NY, United States

**Keywords:** cervical cancer, immunotherapy, checkpoint inhibitor, PD-L1, tumor mutation burden, biomarker

## Abstract

**Background:** Checkpoint inhibitor immunotherapy or immuno-oncology (IO) treatment in refractory cervical cancer yielded an objective response rate (ORR) of 12% in tumors expressing the programmed cell death ligand-1 (PD-L1) in the KEYNOTE-158 phase II study. We hypothesized that the positive response might be associated with the level of PD-L1 expression and/or the tumor mutation burden (TMB). We also aimed to analyze if responses could be associated with platinum sensitivity.

**Methods:** This is a retrospective study of all consecutive patients with cervical cancer who received pembrolizumab or nivolumab.

**Results:** Ten patients were identified. Median age was 64.5 years old (range 48–80). The response rate was 70% and the median duration of response was 21.0 months (range 1.8–26.7) after 20.7 months of follow-up (range 2.0–31.0). The response rate was 80% in patients with PD-L1 combined positive score (CPS) ≥ 10, and 75% in patients with tumor mutation burden (TMB) ≥ 10 mut/Mb. The mean progression-free survival (PFS) for the entire cohort was 20.2 months (95% CI 12.0–28.5). Seven patients had treatment for >12 months (range 14.6–31.0). Five patients were platinum-sensitive and 5 patients were platinum-resistant at the time of immunotherapy, and the response rate was similar regardless of platinum sensitivity.

**Conclusions:** The positive response to IO treatment in advanced cervical cancer in this study was higher than published, and a possible association with the level of PD-L1 expression and the TMB level was suggested. A PD-L1 CPS score ≥ 10 or TMB ≥ 10 may be biomarkers to correlate with response, which should be explored in large studies.

## Introduction

Cervical cancer is the 14th most common cancer among women in the United States of America and is associated with HPV infections (1). Worldwide, it ranks fourth in terms of incidence and mortality among women (2). HPV vaccination and early detection by the Papanicolaou test (also known as the Pap smear) have decreased incidence and promoted early detection (3, 4). Primary treatment with surgery or chemoradiation can cure about 70% of the early stage patients; however, about 30% of the patients ultimately recur after primary treatment with either surgery or definitive chemoradiation (5–7).

Until recently, the treatment for patients with recurrent and metastatic cervical center has been chemotherapy with limited efficacy (8). Combination chemotherapy using cisplatin and topotecan yields a median overall survival of 9.4 months compared to 6.5 months for cisplatin alone (9). Incorporating bevacizumab to the chemotherapy regimen has shown to further increase median overall survival to 16.8 months (10).

A new generation of therapeutics, i.e., immune checkpoint inhibitors or immuno-oncology (IO) treatment, represents a major advance in the treatment of malignancies. Tumor cells evade immune destruction through various ways, such as downregulation of the T cell response and modulation of major histocompatibility antigen expression (11). Immune checkpoint inhibitors are monoclonal antibodies that most commonly target cytotoxic T-lymphocyte antigen 4 (CTLA-4), programmed cell death protein-1 (PD-1), or its ligand (PD-L1) by inhibiting the suppression of T cell activity and re-enabling the immune system to attack tumor cells (12). These therapies have shown antitumor activity in multiple tumor types. The activity of pembrolizumab in cervical cancer has been tested in the phase Ib KEYNOTE-028 and phase II KEYNOTE-158 studies (13, 14). In the latter, the response rate from single agent pembrolizumab was 12% and another 18% had stable disease. In addition, 80% of those who responded had more than 12 months of response. All patients who responded to pembrolizumab had PD-L1 expression of ≥1. Based on those results, pembrolizumab was approved by the FDA for the treatment of advanced cervical cancer that progressed on chemotherapy.

Immune checkpoint inhibitor treatment is emerging as a promising treatment in cervical cancer, but an important unanswered question is the identification of predictive clinical factors or biomarkers associated with treatment response.

In this study, we reviewed our series of cervical cancer patients who received immunotherapy. We aimed to evaluate the correlation of response and progression-free survival with a number of clinical factors, including PD-L1 combined positive score (CPS), tumor mutation burden (TMB), platinum sensitivity, and sites of metastatic disease.

## Methods

This is a retrospective study including all patients with cervical cancer who were treated in Maimonides Cancer Center whose start date of receiving pembrolizumab or nivolumab treatment was before September 31, 2019. The last day of enrollment was August 31, 2019. The study protocol was approved by the Institutional Review Board. Electronic medical records were searched to collect demographics, treatment history and response. Tumor response was assessed by the investigators according to the Response Evaluation Criteria in Solid Tumors (RECIST 1.1) (15). Patients were deemed to be platinum-sensitive if they had previously responded to a platinum-based therapy, and the treatment was given at least 6 months prior. Of note, some patients were given a chemotherapy break after being treated until best response with platinum-based treatment; if their subsequent recurrence was more than 6 months from the last platinum administration, they were deemed to be platinum-sensitive. Platinum-refractory was defined as those patients without a previous response to platinum, or with recurrence <6 months from the last platinum-based treatment. The cut-off day for follow up was April 30, 2021. PD-L1 expression was performed by Foundation Medicine (Cambridge, MA, USA) and Pathline Emerge (Ramsey, NJ, USA). Seven out of 10 patients had data on tumor molecular testing through next-generation sequencing (NGS) performed by Foundation Medicine. Duration of response was defined as time from beginning of response until objective progression or death; PFS was defined as time from start of treatment until objective tumor progression or death; and OS was defined as time from start of treatment until death.

### Statistical Methods

The predictor variables were all coded as binary with dummy variables and included the following: PD-L1 CPS ≥ 10 or PD-L1 CPS < 10, TMB ≥ 10 or TMB < 10, platinum sensitivity or resistance, and tumor site at only the lymph nodes or elsewhere.

Both a Fisher's exact test as well as an unadjusted linear regression were performed to analyze the differences between each of the binary predictor variables. The binary outcome variable in the Fischer's exact test was either a response to treatment or no response to treatment. A Fisher's exact test was performed because of the low cell count and small sample size. The test was first used to calculate the chi-square test statistic and the corresponding *p*-values between the identified predictors and outcome variables. Separate associations were analyzed between the binary outcome for PFS and PD-L1 CPS, TMB, platinum sensitivity, and tumor site. Similar analyses for the same four predictor variables were analyzed for associations with the binary outcome of response to treatment.

Separate unadjusted linear regressions were performed to determine estimates for differences in the continuous variables for PFS and response duration and the predictor variables for PD-L1 CPS, TMB, platinum sensitivity, and tumor site. An estimate in the difference between PFS for those who responded to treatment and those who did not was also analyzed using a linear regression to compare to the results and effectiveness of the treatment in existing literature. Adjusted linear regressions would have resulted in an overfit model because of the small sample size; however, only 2 samples are needed per variable in order to analyze linear regressions with an acceptable degree of internal validity (16). An alpha value of 0.05 and a 95% confidence interval was used to determine statistical significance.

## Results

### Patient Demographics

Ten patients were identified, and their demographics and clinical characteristics are summarized in Table 1 and Supplementary Table 1. Median age was 64.5 years (range 48–80). In terms of ethnic origin, 1 was Caucasian (Russian), 4 were Asian, 3 were Caribbean, and 2 were Latino. All were diagnosed with squamous cell carcinoma, with one transformed to small cell. All patients received platinum-based treatment. Other than 1 patient who had *de novo* metastatic disease, 9 patients had recurrent disease; among them, 7 patients received definitive platinum-based chemoradiation as primary treatment, and 2 patients received adjuvant chemoradiation after surgery. After developing recurrent/metastatic disease, and prior to IO therapy, 8 had received platinum again and 6 had received bevacizumab. At the time of starting IO treatment, 5 patients were still considered to be platinum-sensitive. Their primary sites of disease at the time of IO treatment were lymph node only (LN) (*n* = 3), pelvic organs (*n* = 2), visceral metastasis (*n* = 2), and mixed (*n* = 3).

**Table 1:** Baseline demographics and disease characteristics.

**Disease characteristics**	**Number (*N* = 10)**
**Age, years old**	
Median	64.5
Range	48–80
**ECOG status**	
0	3
1	6
2	0
3	1
**Ethnicity**	
Asian	4
Caribbean	3
Latino	2
Russian	1
**FIGO stage at initial diagnosis**	
I	1
II	5
III	3
IV	1
**Histology**	
Squamous cell	10
Adenocarcinoma	0
Small cell	1 (transformed from squamous cell)
**Previous treatment with platinum**	10
Chemoradiation primary treatment	7
Chemoradiation adjuvant treatment	2
**Chemotherapy for metastatic disease**	
Using platinum	8
Using bevacizumab	6
**No of previous lines of therapy for recurrence/metastasis before IO**	
0	2
1	5
2	1
3	2
**Sites of metastatic lesions**	
LN only	3
Pelvic organs	2
Visceral metastases	2
Other	3
**Platinum sensitivity**	
Sensitive	5
Resistant	5
**Immunotherapy received**	
Pembrolizumab	9
Nivolumab	1
**PD-L1 combined positive score (CPS)**	
0	1
1–9	3
10–100	6
**Microsatellite status (MS)**	
MS stable	7
MS instability	0
Not assessed	3
Tumor mutation burden (TMB)^*^	
TMB <10	3
TMB ≥ 10 mut/Mb	4
**P16 status**	
Positive	6
Negative	1
Not assessed	3

* *Seven of the patients had tumor mutation burden measured*.

Molecular biomarker characteristics were extracted from the tumor genomic testing and pathology reports, shown in Table 1. PD-L1 combined positive score (CPS) was 0 in the patient whose tumor transformed to small cell cancer, 3 (30%) were 1–9, and 6 (60%) were CPS ≥ 10. All had microsatellite stable status. Of the 7 patients with molecular testing, all had TMB ≥ 6, and 4 of these had TMB ≥ 10. P16 was positive for 6, negative for 1.

### Treatment and Response

Nine patients received pembrolizumab and 1 received nivolumab treatment. The best response rate was 70%, with 3 complete response (CR), 4 partial response (PR), 1 stable disease (SD), and 2 progressive disease (PD). One of the two patients with progression was a transformed small cell case. While one patient had PR by clinical imaging, she underwent pelvic surgery and was found to have CR on pathological evaluation.

**Figure 1:**
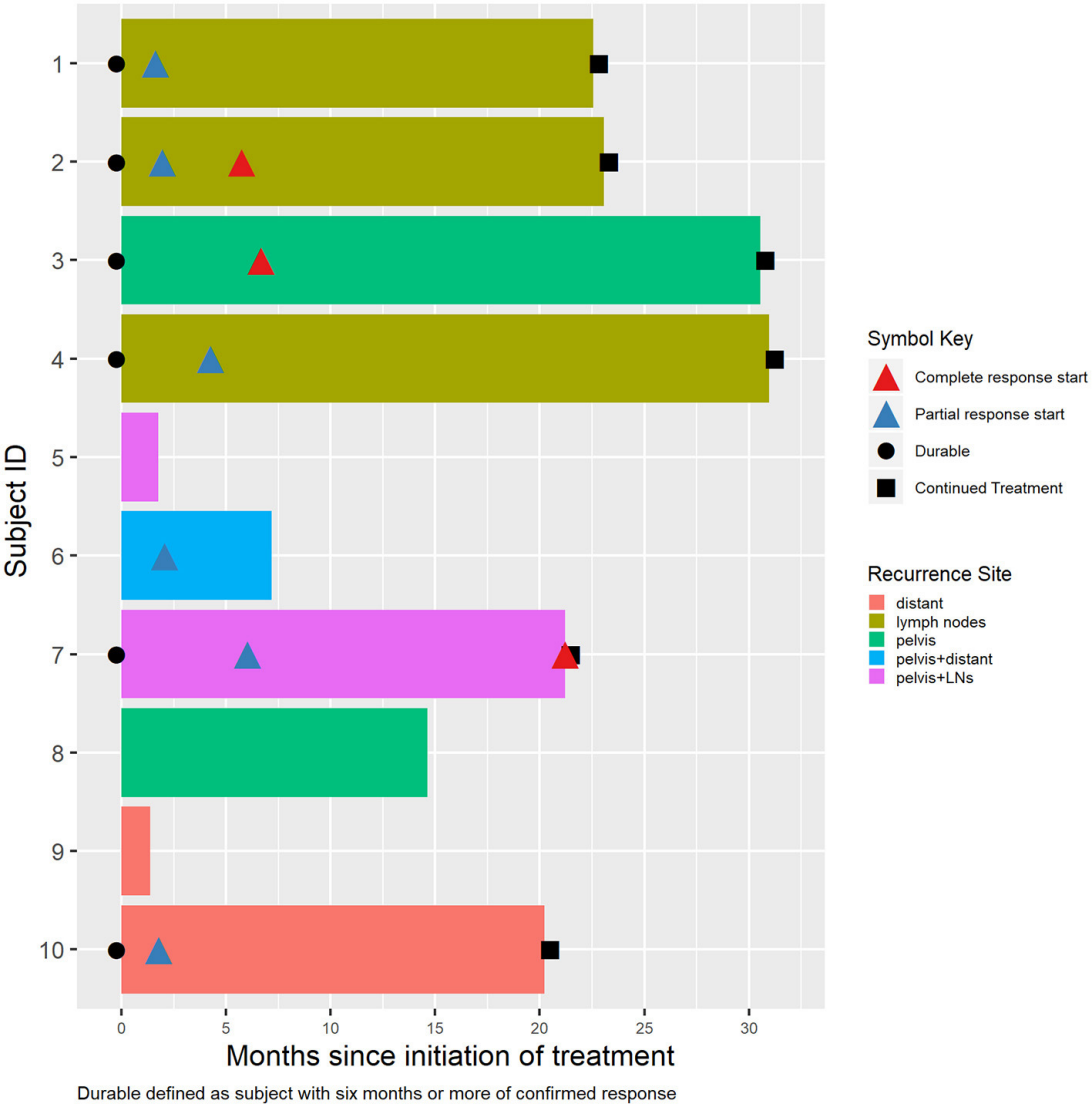
Individual patient disease characteristics, response, and durations.

At the time of data cut-off, the median follow-up was 20.7 months. The median duration of treatment was 26 cycles (range 3–30 cycles) or 20.7 months (range 1.4–31.0 months), and 5 patients were still continuing treatment. One patient stopped treatment after being found to have pathological complete response during pelvic surgery after 21.2 months of treatment. The median duration for treatment for those who responded to treatment was 22.6 months (range 7.2–31.0). Eight patients had continued for >6 months, and 7 had continued for >12 months. Figure 1 depicts patients' best response, duration of response, and unique tumor characteristics.

Both platinum-sensitive patients and platinum-resistant patients demonstrated CR or PR to IO treatment, with response rates of 60 and 80%, respectively.

The mean progression-free survival (PFS) for the entire cohort was 20.2 months (95% CI 12.0–28.5). The mean overall survival (OS) was 21.7 months (95% CI 14.3–29.0) (Figure S1). Only 4 events have happened so we were unable to calculate the median.

### Association of Response With Biomarkers

In the entire cohort, the PD-L1 composite positive score has a median of 10 and a mean of 32.5 (95% CI 7.7–57.3), which indicates a right skew toward the higher expression scores. The overall TMB for the 7 patients included in this measure had a median of 14.0 and a mean of 15.6 (95% CI 5.0–26.1), also skewed toward a higher expression (Supplementary Table 2).

We further attempted to evaluate the association of response in patients with particular biomarker characteristics. Generally, the response rate was higher in patients with CPS ≥ 10 vs. CPS < 10 in PD-L1 expression level (83.3 vs. 50%); higher in TMB ≥ 10 vs. < 10 (75 vs. 33%); and higher in patients with LN only disease vs. non-LN disease (100 vs. 57.1%). It was also higher in patients with platinum refractory disease vs. platinum sensitive disease (80 vs. 60%) (Table 2).

**Table 2:** Correlation of response rate with biomarkers.

	**Response rate**	**Fisher's exact test**
	***N* (%)**	**p-value**
**PD-L1**		0.2598
CPS ≥ 10 (*N* = 6)	5 (83.3)	
CPS <10 (*N* = 4)	2 (50)	
Tumor mutation burden^*^		0.2703
TMB ≥ 10 mut/Mb (*N* = 4)	3 (75)	
TMB <10 mut/Mb (*N* = 3)	1 (33)	
**Platinum sensitivity**		0.4902
Sensitive (*N* = 5)	3 (60)	
Refractory (*N* = 5)	4 (80)	
**Tumor site**		0.1753
Lymph node only (*N* = 3)	3 (100)	
Other [pelvic, visceral, or mixed] (*N* = 7)	4 (57.1)	

* *Seven of the patients had tumor mutation burden measured*.

Using a Fisher's exact test to test the associations between response to treatment and each of the four predictor variables, as summarized in Table 2, the result showed that the associations were >0.05, indicating that at this sample size, there is no detectable statistical significance between response to treatment and PD-L1 CPS, TMB, or platinum sensitivity, or tumor site.

The association of PFS with each of the above four variables was studied using linear regressions method (Table 3). Although not reaching statistical significance, the median and mean PFS for patients with a PD-L1 CPS ≥ 10 was longer than those with a PD-L1 CPS < 10, and the difference in the mean PFS was 3.4 months (95% CI −4.3 to 11.1) longer. Patients with tumors only in the lymph nodes had a mean PFS of about 25.5 months (95% CI 13.8–37.3), which is ~12.9 months (95% CI 6.0–19.8) longer than patients with tumors at pelvic, visceral, or multiple sites. This difference was numerally large, but not statistically significant. However, the 95% confidence interval comparing tumor sites did not include 0, which indicates that there may be a significant difference in PFS given that the study was better powered with a larger sample size (Table 3).

**Table 3:** Correlation of progression-free survival with biomarkers.

**Progression-free survival (months)**	***N***	**Median**	**Mean (95% CI)**	**Parameter estimate (Unadjusted)**	**p-value**
PD-L1 CPS ≥ 10	6	20.68	17.88 (7.45 to 28.31)	3.41 (−4.27 to 11.09)	0.6684
PD-L1 CPS <10	4	12.81	14.46 (−8.74 to 27.67)	Referent	
TMB ≥ 10	4	12.02	12.42 (−4.58 to 29.42)	−7.08 (−17.57 to 3.41)	0.5387
TMB <10	3	19.50	19.50 (−120.97 to 159.97)	Referent	
Platinum-sensitive	5	20.14	15.36 (3.81 to 26.90)	−2.31 (−9.88 to 5.26)	0.7678
Platinum-refractory	5	21.24	17.67 (0.10 to 35.24)	Referent	
Tumor in lymph nodes only	3	23.06	25.54 (13.81 to 37.26)	12.89 (5.95 to 19.84)	0.1006
Other tumor sites	7	8.44	12.64 (2.19 to 23.10)	Referent	
Responded to treatment	7	22.57	21.78 (13.45 to 30.10)	17.55 (23.08 to 12.02)	0.0131^*^
No response to treatment	3	2.56	4.23 −4.91 to 13.36)	Referent	

* *Statistically significant at alpha = 0.05*.

There was no significant difference in the median or mean PFS between patients with platinum-sensitive disease and platinum-refractory disease (Table 3). On the other hand, for patients with TMB < 10, the mean PFS was 7.1 months (95% CI −17.6 to 3.41) longer than patients with TMB ≥ 10.

For patients who responded to treatment, the PFS was significantly longer than those who did not respond to treatment (Table 3).

### Other Mutations

As seven patients had next-generation sequencing for tumor gene profiling, we also examined their common mutations. Among the patients who responded to treatment, PIK3CA mutations were seen in 3 patients, MLL2 mutations were seen in 2, and mutations in the TERT promoter were seen in 2 patients. Those recurrent mutations were not detected in the patients without treatment response. Instead, PALB2, DDR2, and BCL2 amplifications were found (Table S3).

### Adverse Events

Only one patient developed a severe treatment-related immune-associated adverse event while the other patients did not show notable side effects. She had presented with *de novo* metastatic disease with liver, renal and peritoneal metastases. Past medical history included hypertension and ventricular tachycardia and she was taking metoprolol and amiodarone. She was initially treated with paclitaxel and carboplatin for 6 cycles with early response but quick progression. PD-L1 CPS was 60%, so the treatment was switched to pembrolizumab with initial PR. During the response, she progressed to a mixed pattern. Imaging had demonstrated marked decrease of the liver and peritoneal lesions, but enlargement of a kidney lesion. She then developed worsening thrombocytopenia. Treatment was held when the platelet count decreased to 38,000/μL, and eventually reached a nadir of 10,000/μL. She was diagnosed with idiopathic thrombocytopenic purpura (ITP), as her peripheral smear revealed large platelets without clumping. She was admitted for intravenous immunoglobulin (IVIG) and glucocorticoid treatments, and her platelets responded with an increase to 23,000/μL. On day four of the hospital admission, she developed hypoxemia and unresponsiveness, and subsequently expired. Despite the thrombocytopenia, there were no obvious signs of bleeding.

With a median follow-up of 20.7 months, we have not observed other severe immune-related toxicities.

## Discussion

The phase Ib KEYNOTE-028 study and the phase II KEYNOTE-158 study have demonstrated promising antitumor activity with pembrolizumab in patients with advanced cervical cancer who have become refractory to platinum-based chemotherapy (13, 14). However, the overall response rate was reported to be only 12.2%. This notion of low ORR with immunotherapy in this cancer was also shown in a study with nivolumab alone (26.3%) (17), while the combination of nivolumab with ipilimumab appeared to deliver a higher response rate of 46% (18). A significant benefit from immunotherapy is the durable response in the responders (14) (KEYNOTE-158), not only in cervical cancer, but also in other cancers (19, 20).

Only 12 of 98 patients showed overall response in the KEYNOTE-158 study. Although our cohort was smaller, we had a much higher proportion of responders (70%) and a longer follow up time with a median of 20.7 months. Our result should provide addition to the literature regarding the characteristics of the responders.

We have shown in this study that response can occur in both platinum-sensitive or platinum-refractory patients, and in patients with lymph node disease or widespread visceral disease. The treatment response was durable with a median of 21.0 months, which is comparable to published studies. Seven of these patients had responses > 12 months. Our results are consistent with the consensus observations from the vast publications on immunotherapy, in that responding patients may enjoy a long-term control with minimal side effects.

In the published studies, responses appeared to occur in PD-L1 CPS positive patients, but due to the low response rate, more biomarker studies are needed for patient selection and prediction of response. Our study showed a much higher response rate than reported, and it would be interesting to delve deeper into the underlying associations. For example, all our patients were non-US born immigrants. Moreover, the level of expression of PD-L1 had a median of 10 and a mean of 32.5 (95% CI 7.7–57.3), which indicates a right skew (i.e., higher expression). The overall TMB had a median of 14 and a mean of 15.6 (95% CI 5.0–26.1) (Supplementary Table 2), both of which are higher than the median TMB of 5–6 mut/Mb usually found in this disease (21, 22).

In our study, patients with PD-L1 CPS ≥ 10 demonstrated numerically higher response rate than those with CPS < 10, suggesting a higher PD-L1 score could be a biomarker. Such a correlation has also been found in the treatment of lung cancer and esophageal cancer. KEYNOTE-024 investigated non-small cell lung cancer patients who had PD-L1 expression of more than 50% and found that single-agent pembrolizumab induced higher response rates, PFS, and OS than chemotherapy alone (23). Similar treatment advantage was also revealed in patients with esophageal cancer in the KEYNOTE-181 study, (63% of patients with squamous cell histology) in which RR, PFS, and OS all increased with pembrolizumab compared to chemotherapy in patients with PD-L1 expression of at least 10 (24).

The significance of TMB is a rapidly evolving field. In June 2020, the FDA approved pembrolizumab treatment in patients with TMB ≥ 10 mut/Mb regardless of cancer type. The study was based on promising data from the KEYNOTE-158 study, which analyzed a subset of 102 patients (13.2%) whose tumor had a TMB-H signature, defined as TMB ≥ 10 mut/Mb. The ORR was 29% in this study. Among them, 16 patients had cervical cancer with a response rate of 31% (25). The 1-year PFS was also higher in the TMB-H group vs. the non-TMB-H group (26.4 vs. 14.1%, respectively) (26). As mentioned above, the median TMB in cervical cancer was estimated to be 5–6 mut/Mb from prior studies (21, 22); therefore, all our patients had TMB higher than the median. There were 4 patients in our study who had TMB ≥ 10, and their response rate was 75%. These patients would be defined as patients likely having response based on the new approval indication. Thus, the high response rate seen in our cohort could be attributed by the higher proportion of patients with intermediate or high TMB. We propose to further study the relationship of TMB 6–10 mut/Mb and response in future larger studies.

Clinical factors associated with response and PFS were studied. As patients enrolled in the KEYNOTE-158 study were predominantly platinum-refractory patients, our data on platinum-sensitive patients should be supplemental to the literature. Similar response rate and PFS were observed between platinum sensitive and platinum resistant patients. On the other hand, patients with LN-only disease showed a higher response rate, longer mean and median PFS than those with non-LN-only disease, suggesting that patients without hematological spread may fare better with immunotherapy.

We performed statistical analysis attempting to confirm the potential association of biomarkers with response and PFS. There was no statistical significance to satisfy a *p*-value of <0.05, which could be attributable to the small sample size. Furthermore, as our cohort had a relatively higher expression of the biomarkers, which itself may have been the overwhelming basis of the higher response rate, the binary cut off value that was chosen for the comparison between groups may not have been optimal. Nevertheless, the analysis suggests a possible difference in a study with greater power, which encourages further study with larger sample sizes.

Undoubtedly, immunotherapy offers patients a therapeutic option of less toxic treatment with long-term control. The mean PFS of the entire group was 20.2 months, longer than that of the bevacizumab-paclitaxel-cisplatin arm (median PFS of 7.6 months) in a previous chemotherapy study (27). We have not had a chance to observe progression pattern after immunotherapy.

One patient died after developing ITP, which was considered to be IO-related. ITP as a side effect from PD-L1 blockade has been reported in the literature (28), although this complication is uncommon (29). Our patient was diagnosed with grade 4 ITP and treated accordingly and never showed any signs of bleeding. It is unknown if the death was related to the immunotherapy.

## Conclusion

The response rate to IO treatment in cervical cancer was much higher than published data in this small cohort of patients who had TMB ≥ 6. A PD-L1 CPS score ≥ 10 or TMB ≥ 10 may be a biomarker to correlate with response, which should be explored in future large studies.

## Data Availability Statement

The datasets presented in this article are not readily available because they consist of patient records. Requests to access the datasets should be directed to yxu@maimonidesmed.org.

## Ethics Statement

The studies involving human participants were reviewed and approved by the Maimonides Medical Center Institutional Review Board. Written informed consent for participation was not required for this study in accordance with the national legislation and the institutional requirements. Written informed consent was not obtained from the individual(s) for the publication of any potentially identifiable images or data included in this article.

## Author Contributions

YX and AH designed the analysis. KS, YX, and AH performed the analysis and interpreted the results. KS, AH, and YX contributed to the manuscript. All authors approved the submitted version.

## Conflict of Interest

The authors declare that the research was conducted in the absence of any commercial or financial relationships that could be construed as a potential conflict of interest.

## Publisher's Note

All claims expressed in this article are solely those of the authors and do not necessarily represent those of their affiliated organizations, or those of the publisher, the editors and the reviewers. Any product that may be evaluated in this article, or claim that may be made by its manufacturer, is not guaranteed or endorsed by the publisher.
