# Supplementary material for: Response to Immune Checkpoint Inhibitor Treatment in Advanced Cervical Cancer and Biomarker Study
Source: Front Med (Lausanne). 2021 Aug 12;8:669587. doi: 10.3389/fmed.2021.669587 (PMC8387671; doi:10.3389/fmed.2021.669587)
Supplement: Supplementary file 1 [file Data_Sheet_1.docx]

Supplementary Material

# Supplementary Tables

|  | Initial treatment with platinum: primary or adjuvant | Initial Recurrent site | Initial treatment for recurrence | XRT for curative intent for first recurrence of disease | 2^nd^ recurrence site | 2^nd^ line treatment | 3^rd^ line treatment | 4^th^ line treatment | Platinum sensitive or resistant at the time of immunotherapy | Best response | Duration of response (m)* |
| --- | --- | --- | --- | --- | --- | --- | --- | --- | --- | --- | --- |
| 1 | Primary | External iliac LN | Immuno |  |  |  |  |  | Sensitive | PR | 20.96 |
| 2 | Primary | Para aortic, mediastinal LN | Avastin, Abraxane, cisplatin |  | Para aortic LN | Immuno |  |  | Sensitive | CR | 21.13 |
| 3 | Adjuvant | Vaginal cuff | Avastin, Abraxane, cisplatin | yes | Vaginal cuff, to the pelvic side wall | Avastin, Taxol, topotecan | Immuno |  | Resistant | CR | 23.89 |
| 4 | Primary | Bilateral lower neck, mediastinal LN | Avastin, Taxol, carboplatin |  | Left supraclavicular, mediastinal | Avastin and Taxol | Cisplatin and topotecan | Immuno | Resistant | PR | 26.74 |
| 5 | Adjuvant | Para aortic | Avastin, Taxol, carboplatin | yes | Axillary, subcarinal, peritoneal | Immuno |  |  | Sensitive | PD | N/A |
| 6 | Primary, metastatic | De novo hepatic mets | Taxol and carboplatin |  | Liver, pelvis | Immuno |  |  | Resistant | PR | 1.84 |
| 7 | Primary | cervix | Cisplatin and Taxol |  | Pelvic mass | Immuno |  |  | Resistant | CR | 15.21 |
| 8 | Primary | Vaginal wall | Immuno |  | Vaginal wall, pelvis |  |  |  | Sensitive | PD | N/A |
| 9 | Primary | Retroperitoneal mass | Carboplatin and Taxol | yes | Liver | Avastin and Taxol | Carboplatin and etoposide | Immuno (nivolumab) | Resistant | PD | N/A |
| 10 | Primary | Axillary LNs, multiple intramuscular lesions | Avastin, Taxol, cisplatin |  | Liver, left kidney | Immuno |  |  | Sensitive | PR | 18.37 |

Table S1. Clinical characteristics and treatment trajectory of patients treated with immunotherapy

Abbreviations: Immuno: immunotherapy with PD-L1 inhibitor

*N/A: not applicable; duration of response is not calculable for patients with disease progression

| **Variable** | **Median** | **Mean (95% CI)** |
| --- | --- | --- |
| Progression-free survival | Not reached | 20.25 (12.04-28.46) |
| Overall survival | Not reached | 21.66 (14.28-29.05) |
| Response duration | 20.24 | 17.36 (9.62-25.09) |
| PD-L1 Combined Positive Score | 10.00 | 32.5 (7.72-57.28) |
| Tumor mutation burden | 14.00 | 15.57 (5.04-26.10) |

Table S2. Calculated values for given variables

| Patient | PD-L1 | TMB | Mutations |
| --- | --- | --- | --- |
| 1 | 100 |  |  |
| 2 | 5 | 16 | PIK3CA E545K, MLL2 G5181E |
| 3 | 3 | 9 | PIK3CA E542K, ARID1A P408L, BAP1 Q36*, CDKN2A/B loss, CREBBP E1766*, MTAP loss exons 2-8 |
| 4 | 65 |  |  |
| 5 | 2 | 40 | PAL82 M1?, DDR2 D394H |
| 6 | 60 | 15 | FGFR2 P253R, PIK3CA E542K/E726K/M1004I, PTEN Q171*/S287*-subclonal, SMARCB1 R377H, CASP8 Q482*, MAP3K1 Q624*, MAP3K13 E780K*-subclonal, MLL2 Q4085*/S2312*, TERT promoter -164C>T |
| 7 | 10 |  |  |
| 8 | 50 | 6 | FBXW7 R658* |
| 9 | 0 | 9 | BCL2 amp |
| 10 | 30 | 14 | AKT1 E17K, KRAS G13D, MAPK1 D321N, TERT promoter -124C>T |

Table S3. Patients and measured biomarkers

# Supplementary Figures


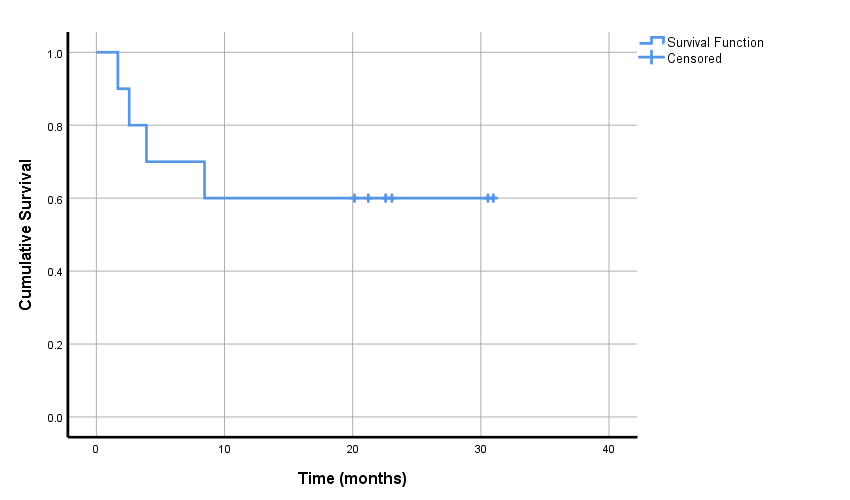

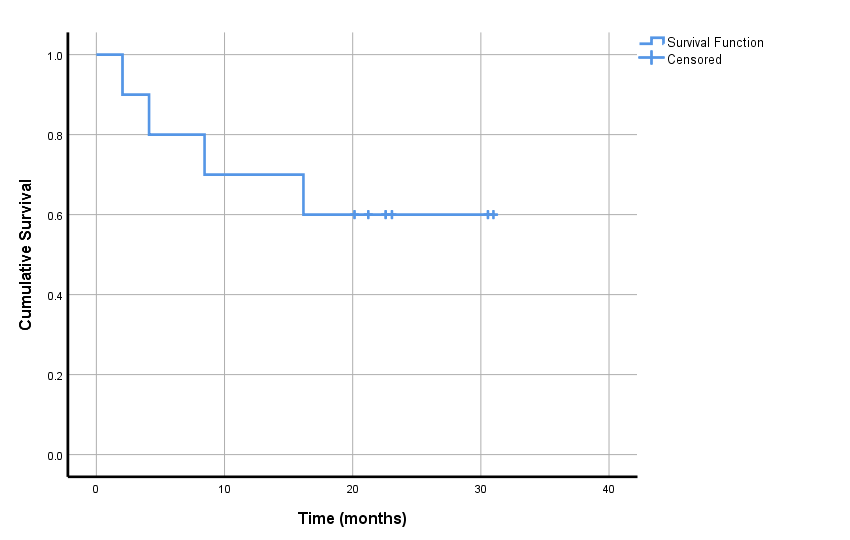


# (a) (b)

Figure S1. Kaplan-Meier survival plots. (a) Overall survival; (b) Progression-free survival
